# Pharmacological Elevation of Cellular Dihydrosphingomyelin Provides a Novel Antiviral Strategy against West Nile Virus Infection

**DOI:** 10.1128/aac.01687-22

**Published:** 2023-03-15

**Authors:** Nereida Jiménez de Oya, Ana San-Félix, Mireia Casasampere, Ana-Belén Blázquez, Patricia Mingo-Casas, Estela Escribano-Romero, Eva Calvo-Pinilla, Teresa Poderoso, Josefina Casas, Juan-Carlos Saiz, María-Jesús Pérez-Pérez, Miguel A. Martín-Acebes

**Affiliations:** a ZOOVIR, Department of Biotechnology, Instituto Nacional de Investigación y Tecnología Agraria y Alimentaria (INIA), Consejo Superior de Investigaciones Científicas (CSIC), Madrid, Spain; b Instituto de Quimica Medica (IQM), CSIC, Madrid, Spain; c Department of Biological Chemistry, Institute for Advanced Chemistry of Catalonia (IQAC), CSIC, Barcelona, Spain; d Liver and Digestive Diseases Networking Biomedical Research Centre (CIBEREHD), Instituto de Salud Carlos III (ISCIII), Madrid, Spain

**Keywords:** West Nile virus, flavivirus, antiviral, sphingolipid, polyphenol, antiviral agents, polyphenols

## Abstract

The flavivirus life cycle is strictly dependent on cellular lipid metabolism. Polyphenols like gallic acid and its derivatives are promising lead compounds for new therapeutic agents as they can exert multiple pharmacological activities, including the alteration of lipid metabolism. The evaluation of our collection of polyphenols against West Nile virus (WNV), a representative medically relevant flavivirus, led to the identification of *N*,*N*′-(dodecane-1,12-diyl)bis(3,4,5-trihydroxybenzamide) and its 2,3,4-trihydroxybenzamide regioisomer as selective antivirals with low cytotoxicity and high antiviral activity (half-maximal effective concentrations [EC_50_s] of 2.2 and 0.24 μM, respectively, in Vero cells; EC_50_s of 2.2 and 1.9 μM, respectively, in SH-SY5Y cells). These polyphenols also inhibited the multiplication of other flaviviruses, namely, Usutu, dengue, and Zika viruses, exhibiting lower antiviral or negligible antiviral activity against other RNA viruses. The mechanism underlying their antiviral activity against WNV involved the alteration of sphingolipid metabolism. These compounds inhibited ceramide desaturase (Des1), promoting the accumulation of dihydrosphingomyelin (dhSM), a minor component of cellular sphingolipids with important roles in membrane properties. The addition of exogenous dhSM or Des1 blockage by using the reference inhibitor GT-11 {*N*-[(1*R*,2*S*)-2-hydroxy-1-hydroxymethyl-2-(2-tridecyl-1-cyclopropenyl)ethyl]octanamide} confirmed the involvement of this pathway in WNV infection. These results unveil the potential of novel antiviral strategies based on the modulation of the cellular levels of dhSM and Des1 activity for the control of flavivirus infection.

## INTRODUCTION

Polyphenols are secondary metabolites widely distributed in all higher plants, which have important roles in defense against pathogenic microbes and herbivores and serve to protect plants from several environmental stresses such as rainfall and UV radiation (1). They are also responsible for flower coloration (2). Over 8,000 polyphenols have been identified until now (3). They are organized into different families according to their structures, with the phenolic acids being one of the most representative (4). A member of this group is gallic acid (3,4,5-trihydroxybenzoic acid), a low-molecular-weight triphenol abundantly present in free or ester forms in plants and also in different vegetable foods such as grapes, nuts, and berries, etc., and beverages such as wine, coffee, and tea (5). Gallic acid and its esters (gallates) have shown preventive and therapeutic effects in many diseases where oxidative stress has been involved, including cancer (6), cardiovascular diseases (7), neurodegenerative disorders (8), and aging (9). In addition, many antimicrobial activities, such as antibacterial (10), antifungal (11), and antiviral (12–16) activities, have been described for this family of polyphenols. Due to this, gallic acid and its derivatives are considered promising lead compounds for new therapeutic agents (17). Our research group has synthesized alkyl esters and amides of gallic acid and its regioisomer 2,3,4-trihydroxybenzoic acid. These compounds displayed antiviral activity against human hepatitis C virus (HCV), a single-stranded RNA virus belonging to the genus *Hepacivirus* within the family *Flaviviridae*. Preliminary data suggested that the antiviral effect of our galloyl derivatives on HCV could be related to alterations in cellular lipids (18), although the specific mechanism was not elucidated.

The family *Flaviviridae* also includes arthropod-borne pathogens transmitted by mosquitos or ticks classified into the genus *Flavivirus*, which contains more than 50 species (19). Vector-borne flaviviruses are responsible for a variety of human and animal illnesses (20). West Nile virus (WNV) and Usutu virus (USUV) cause neurological diseases (21, 22), yellow fever virus and dengue virus (DENV) cause hemorrhagic fevers (23), and Zika virus (ZIKV) is responsible for birth defects and an autoimmune disease (Guillain-Barré syndrome) with neurological symptoms (24). In a manner similar to that observed for other arthropod-borne viruses (arboviruses), the impact of flaviviruses on human and animal health has increased during the last decades due to a variety of factors, which include climate warming, globalization of travel and trade, changes in land use and urbanization, and alterations in vector behaviors (20, 25). At present, there is no licensed specific therapy against any flavivirus, and only a few preventive vaccines are available. Therefore, the need for new antiviral therapies is a priority to combat these pathogens. Among antiviral strategies under investigation, host-targeted antivirals have been raised as feasible alternatives to combat viral infections, in contrast to specific antivirals targeting viral components (26). Their theoretical advantages include their potential broad-spectrum activity against present and future viral threats that share a dependence on host factors and the high genetic barrier to the development of viral resistance (27, 28). In this scenario, growing evidence supports the druggability of lipid metabolism to inhibit viral infections (29, 30). In the case of flaviviruses, infection is strictly dependent on specific lipids required for intracellular membrane rearrangements to establish viral replication factories, energy metabolism, innate immune evasion, and particle biogenesis (31). In fact, certain sphingolipids, such as ceramide (Cer) and sphingomyelin (SM), play crucial roles in flavivirus infection (32–34).

We have evaluated the antiviral potential of a series of polyphenols in infection with WNV, selected here as a representative medically relevant flavivirus. Our results indicate that these compounds reduce WNV multiplication by altering sphingolipid metabolism due to the inhibition of Cer desaturase (Des1), leading to the accumulation of dihydrosphingomyelin (dhSM), a minor component of cellular sphingolipids with important roles in membrane properties. Consistent with the broad-spectrum antiviral potential of polyphenols (35), these compounds also inhibited the multiplication of the clinically relevant related flaviviruses USUV, DENV-2, and ZIKV. Overall, these results highlight the potential of synthetic polyphenols for the development of broad-spectrum antiviral therapies and unveil a novel antiviral strategy against flaviviruses by interfering with the cellular levels of dhSM.

## RESULTS

### Antiviral activity of gallic acid derivatives against WNV.

**FIG 1:**
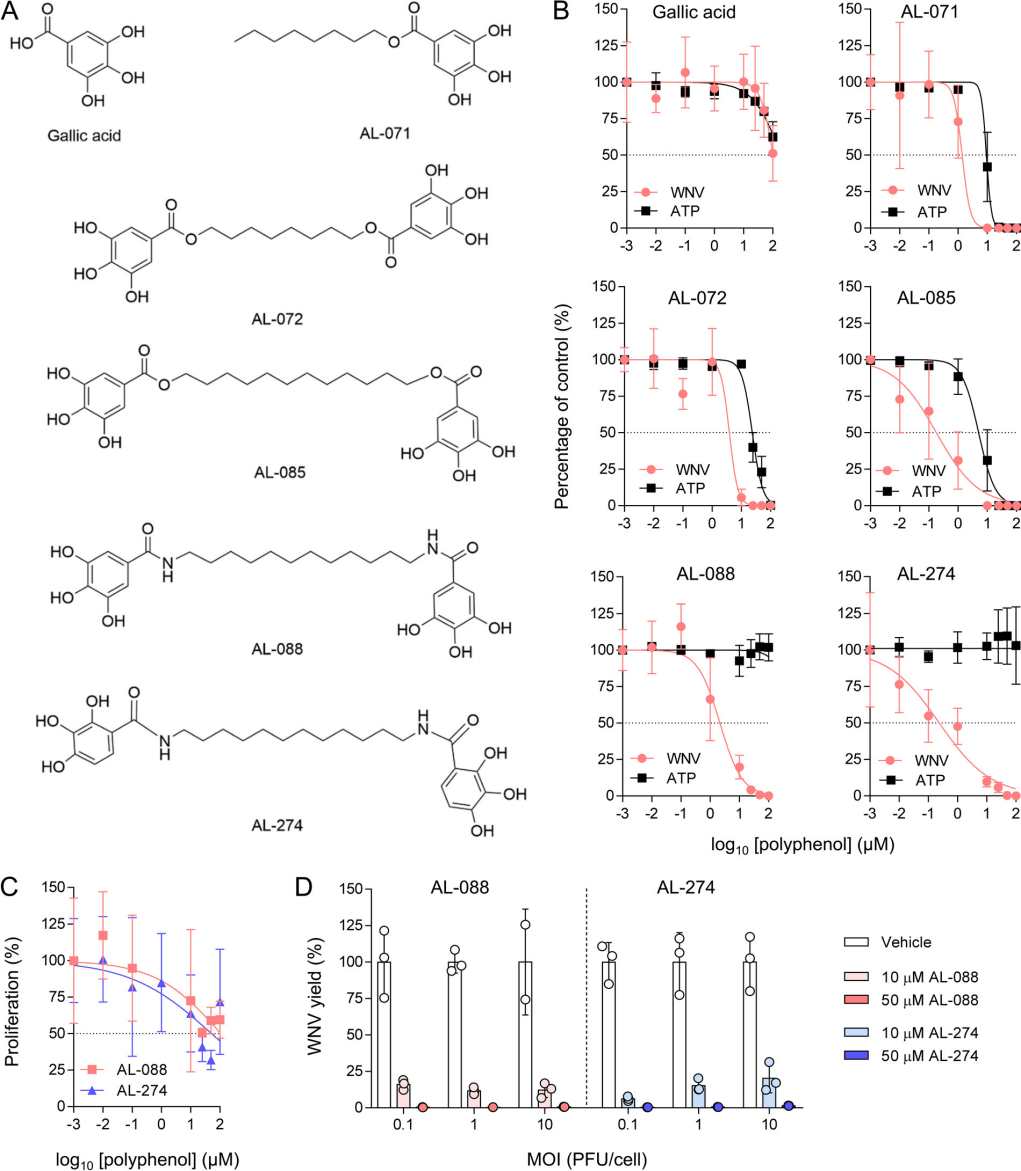
Antiviral activity of synthetic polyphenols against WNV in Vero cells. (A) Compounds used in this study. (B) Antiviral activity against WNV and cytotoxicity of polyphenols. The virus yield in the supernatant of infected Vero cells (MOI of 1 PFU/cell) was determined at 24 h p.i. Polyphenols were added 1 h prior to infection and maintained throughout the rest of the assay. The cytotoxicity of the compounds was measured by the determination of the cellular ATP concentration in uninfected samples. The dashed lines denote a 50% reduction. (C) Effect of polyphenols on the proliferation of Vero cells. Vero cells plated at a low density (<50% confluence) were incubated for 24 h in the presence of the compounds, and the cell number was determined. The dashed lines denote a 50% reduction. (D) Effect of the MOI on the antiviral activity of polyphenols. Vero cells were treated and infected at different MOIs as described above for panel A. Each dot denotes a single biological replicate. Data are expressed as means ± SD (*n* = 2 to 4).

**FIG 2:**
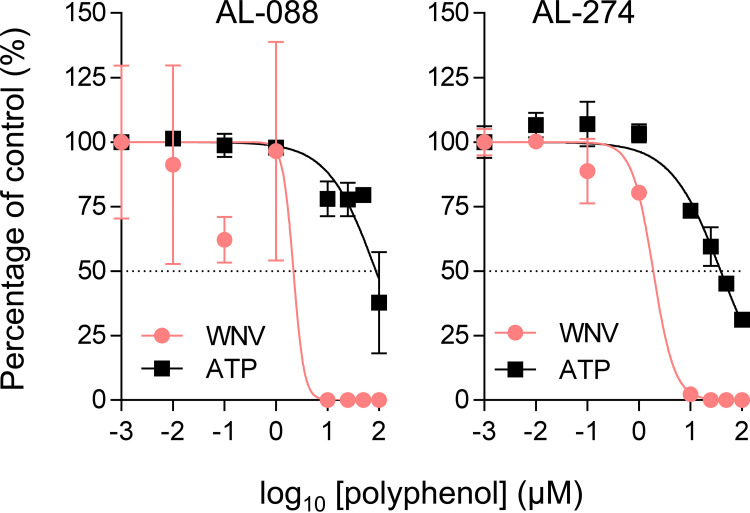
Antiviral activity of synthetic polyphenols against WNV in SH-SY5Y cells. The virus yield in the supernatant of infected SH-SY5Y cells (MOI of 1 PFU/cell) was determined at 24 h p.i. Polyphenols were added 1 h prior to infection and maintained throughout the rest of the assay. The cytotoxicity of the compounds was measured by the determination of the cellular ATP concentration in uninfected samples. The dashed lines denote a 50% reduction. Data are expressed as means ± SD (*n* = 2 to 4).

The structures of the compounds under study are shown in Fig. 1A and include gallic acid; the octyl ester of gallic acid (AL-071); the esters AL-072 and AL-085, containing an octyl or dodecyl methylene linker connecting two subunits of gallic acid at the ends; and the amides AL-088 and AL-274, containing a dodecyl methylene linker and two galloyl moieties (AL-088) or its 2,3,4-trihydroxybenzoyl isomeric ring, respectively, at both ends (AL-274). The antiviral activities of these compounds against WNV in Vero cells, together with their effect on cell viability estimated by ATP measurements, are shown in Fig. 1B. Values of the virus yields in PFU per milliliter are shown in Table S1 in the supplemental material. Table 1 summarizes the half-maximal effective concentrations (EC_50_s), half-maximal cytotoxic concentrations (CC_50_s), and selectivity indices (SIs) of these compounds. Gallic acid showed a slight reduction in the WNV yield concomitant with a reduction in cellular viability, suggesting that the effect on virus growth was due to cytotoxicity rather than specific antiviral activity. The introduction of an acyl chain containing 8 methylenes resulted in the alkyl gallate AL-071 showing some antiviral activity but also higher cytotoxicity. The incorporation of a second gallic unit at the distal position of the octyl spacer (compound AL-072) did not improve the antiviral efficacy. However, when the linker between both gallic acid units was extended to a dodecyl unit (AL-085), the antiviral efficacy was significantly improved. Even more, the replacement of ester bonds with amides between the galloyl or 2,3,4-trihydroxybenzoyl units and the dodecyl methylene linker gave compounds AL-088 and AL-274, which resulted in significant antiviral activity against WNV (EC_50_ values in the low-micromolar range) and greatly reduced cytotoxicity, conferring remarkable selectivity indices. In addition, the presence of amide bonds linking the distal polyphenols with the polymethylene chain should also improve the metabolic stability versus esterases. AL-088 and AL-274 exerted only cytostatic effects on Vero cells at high concentrations, further supporting their low-cytotoxicity profiles (Fig. 1C). The antiviral activities of AL-088 and AL-274 were very similar despite the multiplicity of infection (MOI) used in these experiments (Fig. 1D and Table S1). As the central nervous system is a major target for WNV replication, the antiviral activity of compounds AL-088 and AL-274 was evaluated in SH-SY5Y cells, a human neuroblastoma cell line susceptible to WNV infection (36). Both compounds exhibited potent antiviral activity (EC_50_ values of 2.2 and 1.9 μM for AL-088 and AL-274, respectively), retaining good selectivity indices (Fig. 2, Table 1, and Table S1). Therefore, compounds AL-088 and AL-274 were advanced for further studies to evaluate their antiviral properties.

**TABLE 1:** Antiviral efficacy and cytotoxicity of polyphenols

Parameter	Value for compound							
	Vero cells						SH-SY5Y cells	
	Gallic acid	AL-071	AL-072	AL-085	AL-088	AL-274	AL-088	AL-0274
CC_50_ (μM) in uninfected cells^*a*^								
24-h treatment	>100	9.2	23.2	5.0	>100	>100	87.6	38.9
48-h treatment					>100	>100		
EC_50_ (μM)								
WNV	~100	1.4	3.9	0.2	2.2	0.24	2.2	1.9
USUV					0.8	1.3		
ZIKV					6.7	2.1		
DENV-2					7.2	9.2		
VSV					25.3	43.0		
CVB5					>100	>100		
SI (CC_50_/EC_50_)								
WNV	>1.0	6.6	5.9	25.0	>45.0	>416	39.8	20.5
USUV					>125	>76.9		
ZIKV					>14.9	>47.6		
DENV-2					>13.9	>10.8		
VSV					>4.0	>2.3		
CVB5					1	1		

a Data are expressed in micromolar and correspond to the means (*n* = 2 to 4).

### Antiviral spectrum of AL-088 and AL-274.

**FIG 3:**
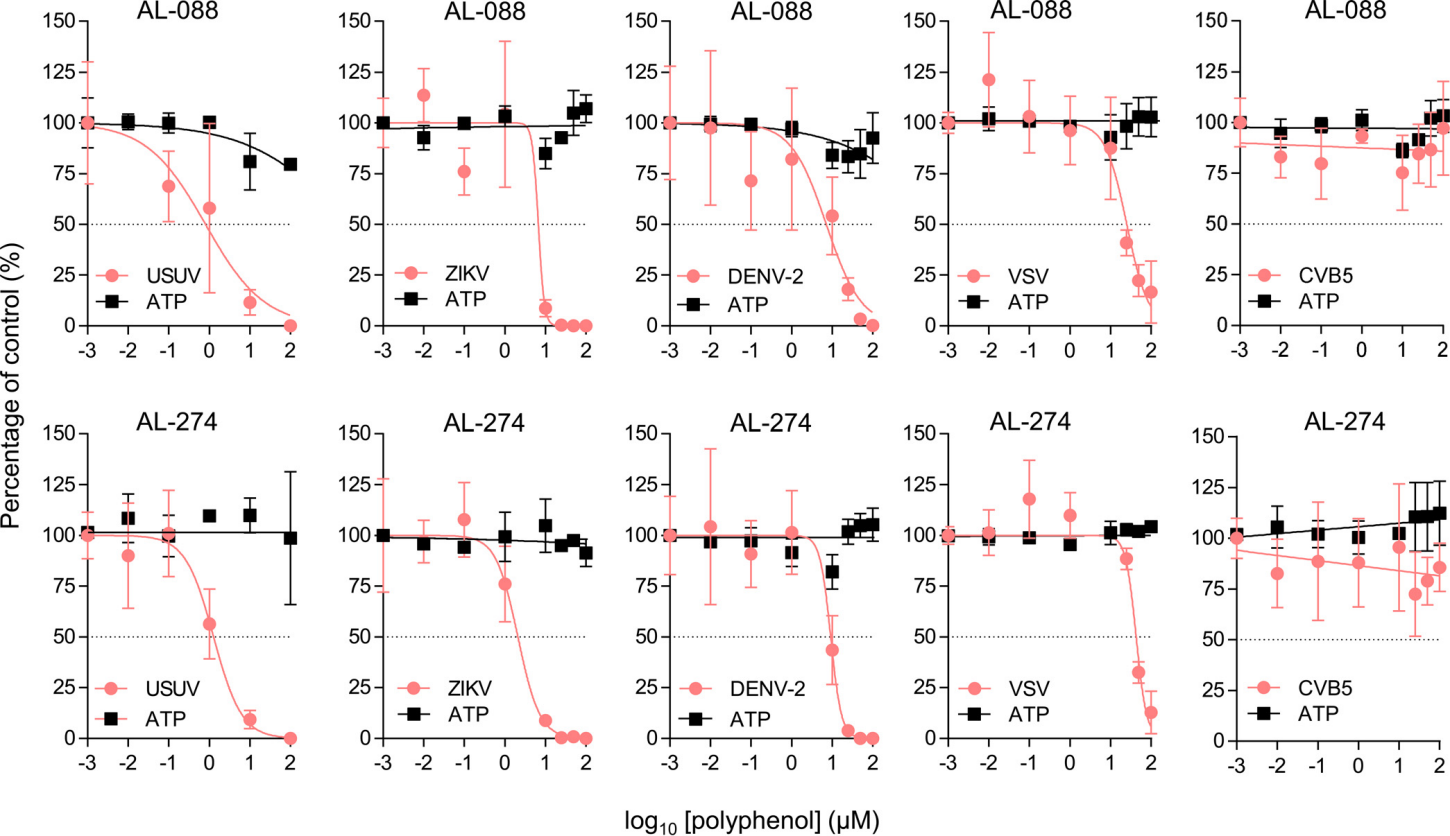
Antiviral spectrum of AL-088 and AL-274. The virus yield in the supernatant of infected Vero cells (MOI of 1 PFU/cell) was determined at 24 h p.i. for USUV, ZIKV, VSV, and CVB5 and at 48 h p.i. in the case of DENV-2. Polyphenols were added 1 h prior to infection and maintained throughout the rest of the assay. The cytotoxicity of the compounds was measured by the determination of the cellular ATP concentration in uninfected samples at 48 h for DENV-2 and 24 h for the rest of the viruses. The dashed lines denote a 50% reduction. Data are expressed as means ± SD (*n* = 2 to 4).

Compounds AL-088 and AL-274 also showed potent antiviral activities against the flaviviruses USUV, ZIKV, and DENV-2 (Fig. 3, Table 1, and Table S1), which were especially remarkable against USUV, with EC_50_ values of 0.8 and 1.3 μM, respectively. However, their antiviral efficacy was reduced against vesicular stomatitis virus (VSV), an RNA virus from the family *Rhabdoviridae*, or even undetectable against coxsackievirus B5 (CVB5), a representative member of the genus *Enterovirus* of the family *Picornaviridae*, pointing to the selective inhibition of flaviviruses.

### AL-088 and AL-274 inhibit WNV multiplication and infectivity.

**FIG 4:**
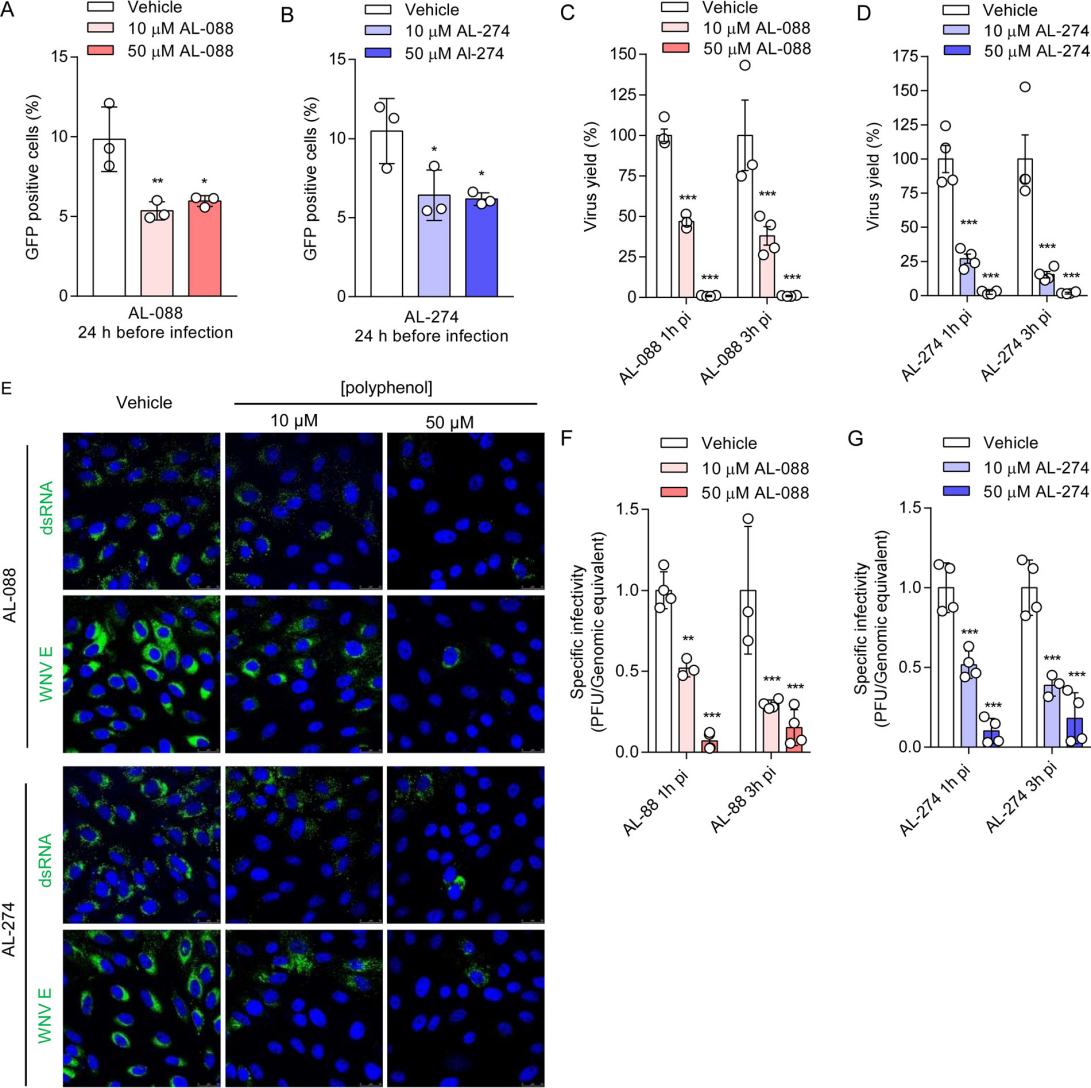
AL-088 and AL-274 inhibit WNV multiplication and infectivity. (A and B) Synthetic polyphenols inhibit the entry of WNV RVPs. Vero cells were treated with AL-088 (A) or AL-274 (B) for 24 h and then infected with RVPs. The number of cells infected with RVPs was determined at 48 h p.i. by flow cytometry (*n* = 3). (C and D) Synthetic polyphenols inhibit WNV multiplication when added at postentry steps. Vero cells were infected with WNV (MOI of 1 PFU/cell), and AL-088 (C) or AL-274 (D) was added at 1 or 3 h p.i. The virus yield was determined at 24 h p.i. (*n* = 6). (E) Gallic acid derivatives inhibit the accumulation of dsRNA intermediates and viral proteins. Vero cells were infected with WNV, treated with the polyphenols at 3 h p.i., fixed, and processed for immunofluorescence to detect dsRNA or WNV E at 24 h p.i. Viral antigens are displayed in green, and cell nuclei are shown in blue. (F and G) Analysis of the specific infectivity of the viral progeny released from cells treated with AL-088 (F) or AL-274 (G) as described above for panels A and B (*n* = 3 to 4). For panels A to D, F, and G, data are expressed as means ± SD. Each dot denotes a single biological replicate. **, *P < *0.01; ***, *P < *0.001 (by ANOVA and Student’s *t* test using Bonferroni’s correction).

Reporter virus particles (RVPs), produced by the *trans*-complementation of a subgenomic flavivirus replicon with an expression vector encoding structural proteins, provide a single-cycle infectious system useful for evaluating the antiviral activity of entry inhibitors (37, 38). Accordingly, the effect of AL-088 and AL-274 on virus entry was analyzed using RVPs (39). Interestingly, pretreatment with the compounds significantly reduced the number of infected cells, indicating that the compounds diminished the entry of RVPs (Fig. 4A and B). To analyze the ability of these compounds to inhibit viral multiplication, cells were infected with WNV and treated with the polyphenols at 1 or 3 h postinfection (p.i.) (Fig. 4C and D and Table S1). AL-088 and AL-274 significantly affected WNV infection when added at 1 or 3 h p.i., suggesting that both compounds could also affect postentry steps. Cells infected and treated with the polyphenols were observed by confocal microscopy (Fig. 4E). A reduction in the accumulation of both double-stranded RNA (dsRNA) intermediates (which constitute reliable markers of flavivirus replication) and the WNV envelope (E) glycoprotein was noticed. Flavivirus replication and particle biogenesis take place coupled in the same membranous structures derived from the endoplasmic reticulum (ER) of the infected cell. Therefore, to analyze the potential effect of the treatment on the biogenesis and infectivity of the viral progeny, we compared the specific infectivities of the viral particles released from cells treated with AL-088 and AL-274 (Fig. 4F and G). The released WNV particles produced by the infected cells after treatment with AL-088 and AL-274 showed a significant reduction in their infectivity compared to the vehicle (Fig. 4F and G), supporting that the compounds could also interfere with the morphogenesis of infectious particles. Taken together, these results suggested that galloyl derivatives exerted their antiviral effect at multiple steps of the viral replication cycle.

### AL-088 and AL-274 elevate dihydrosphingomyelin levels.

**FIG 5:**
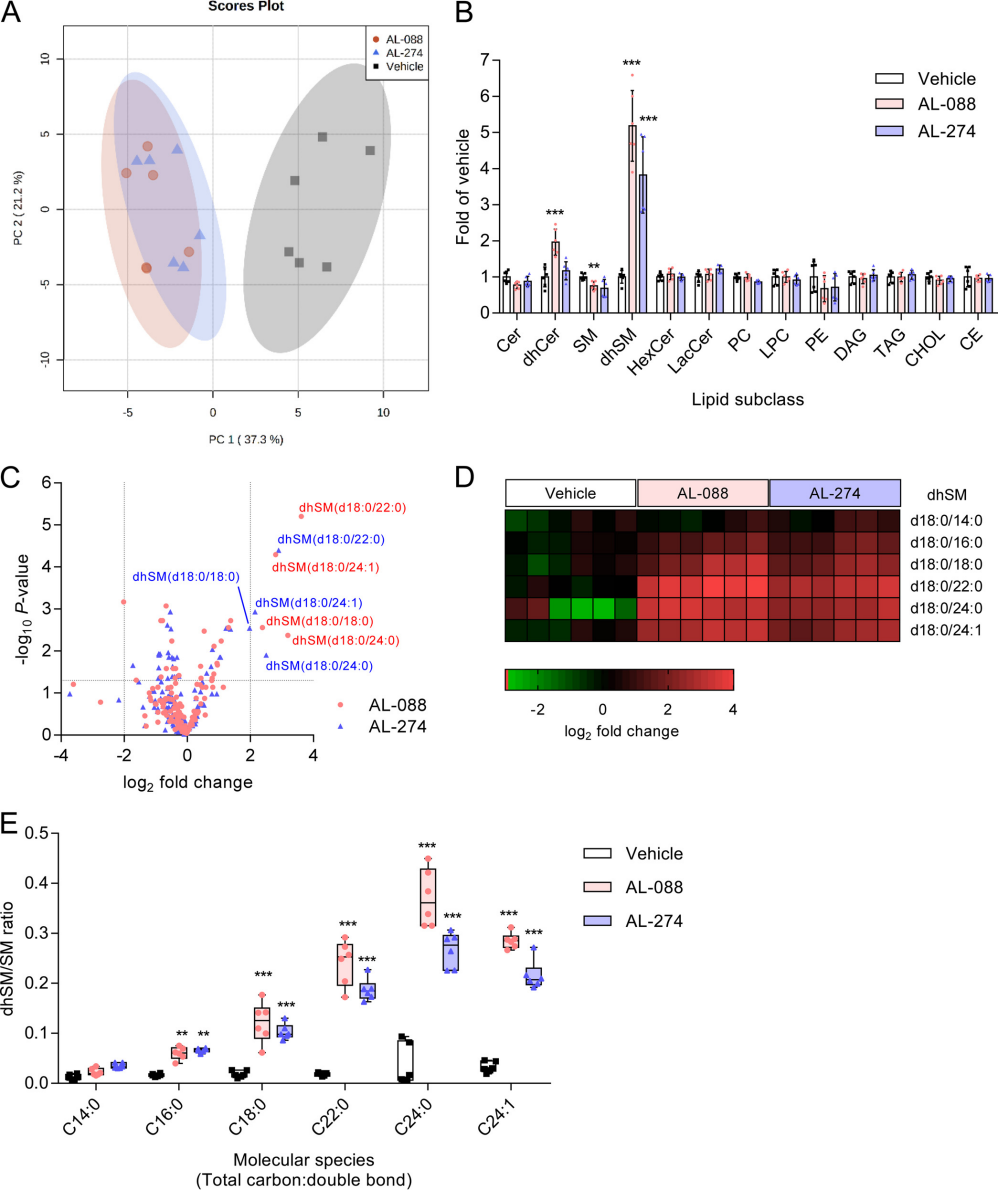
AL-088 and AL-274 elevate dihydrosphingomyelin levels. (A) Comparison of the lipidomes of Vero cells treated with 10 μM polyphenols (24 h) or the vehicle by the PCA method. Each dot denotes a single biological replicate. The 95% confidence regions for each group of samples are colored (*n* = 6). (B) Relative abundances of ceramide (Cer), dihydroceramide (dhCer), sphingomyelin (SM), dihydrosphingomyelin (dhSMs), hexosylceramide (HexCer), lactosylceramide (LacCer), phosphatidylcholine (PC), lysophosphatidylcholine (LPC), phosphatidylethanolamine (PE), diacylglycerol (DAG), triacylglycerol (TAG), cholesterol (CHOL), and cholesteryl ester (CE) in samples treated with AL-088, AL-274, or the vehicle. Each symbol denotes a single biological replicate (*n* = 6). **, *P < *0.01; ***, *P < *0.001 (by multiple *t* tests applying Sidak-Bonferroni correction). (C) Volcano plot displaying the lipid species significantly altered in cells treated with AL-088 and AL-274. Discontinuous lines indicate an FDR-adjusted *P* value of 0.5 and a log_2_ fold change of 2. Each point corresponds to the mean value obtained for a single lipid species (*n* = 6). (D) Heat map displaying the relative abundances of dhSM species in samples treated with the vehicle, AL-088, or AL-274. Each column denotes a single biological replicate (*n* = 6). (E) Box-and-whisker plots showing the dhSM/SM ratios for each molecular species analyzed. Each symbol denotes a single biological replicate (*n* = 6). **, *P < *0.01; ***, *P < *0.001 (by multiple *t* tests applying Sidak-Bonferroni correction).

As mentioned above, flavivirus replication is highly dependent on cellular lipid metabolism, and previous studies supported the potential interference of AL-274 with cellular lipids (18). Therefore, we considered it of particular interest to investigate whether the antiflavivirus activity shown by AL-088 and AL-274 could be due to an alteration in cellular lipid metabolism. With this aim, we evaluated the changes in the lipidomes of cells treated with AL-088 and AL-274. In these analyses, 145 different molecular species belonging to 13 different lipid classes were identified (Fig. S1). Multivariate analyses based on principal-component analysis (PCA) indicated that both AL-088 and AL-274 altered the content of cellular lipids in similar manners (Fig. 5A). This was evidenced by the high degree of overlap between the two groups of samples treated with either AL-088 or AL-274, which clearly differed from those treated with the vehicle only (Fig. 5A). By dissecting data at the lipid subclass level, a significant increase in the level of dihydrosphingomyelins (dhSMs) was noticed in samples treated with AL-088 or AL-274 relative to the control (Fig. 5B). Significantly elevated levels of dihydroceramides (dhCers) and slight reductions in the levels of sphingomyelins (SMs) were also detected in samples treated with AL-088, further supporting the effect of this polyphenol on sphingolipid metabolism. At the molecular species level, only dhSMs (d18:0/18:0, d18:0/22:0, d18:0/24:0, and d18:0/24:1) were significantly altered in samples treated with AL-088 and AL-274 (Fig. 5C). This increase can be clearly visualized in the heat map displayed in Fig. 5D. Compared to the vehicle-treated samples, the dhSM/SM ratio was significantly increased for almost all of the dhSMs analyzed, confirming the enrichment of dhSMs in cells treated with AL-088 and AL-274 (Fig. 5E). These results indicate that these galloyl derivatives significantly alter sphingolipid metabolism and promote dhSM accumulation.

### AL-088 and AL-274 inhibit ceramide desaturase.

**FIG 6:**
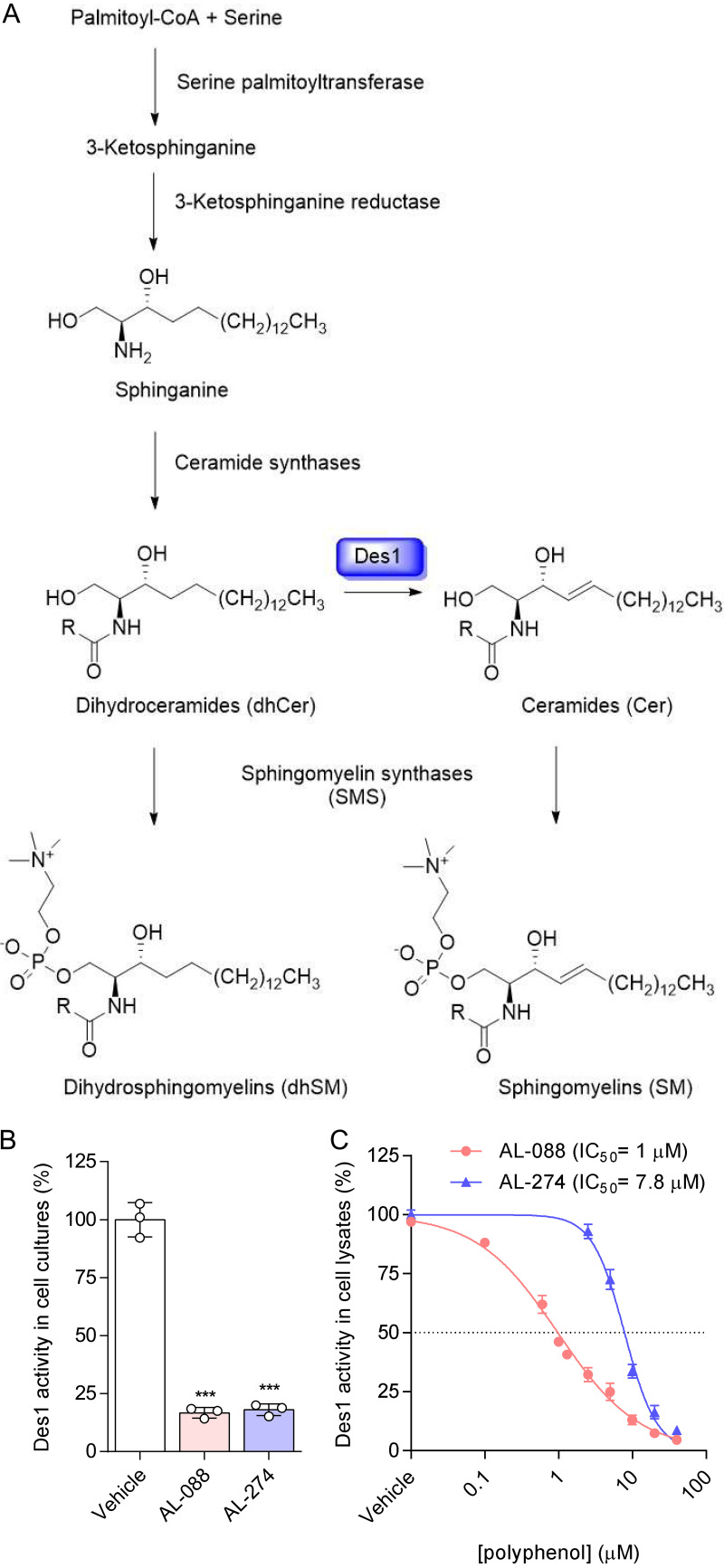
AL-088 and AL-274 inhibit ceramide desaturase activity. (A) Scheme of dihydrosphingolipid synthesis. Ceramide desaturase (Des1) is highlighted in blue. (B) Effect of AL-088 and AL-288 on the conversion of dhCerC6NBD to CerC6NBD in intact T98G cell cultures. Cells were treated with 10 μM each compound. Data are expressed as means ± SD. Each symbol denotes a single biological replicate (*n* = 3). ***, *P < *0.001 (by ANOVA and Student’s *t* test using Bonferroni’s correction). (C) Dose-dependent inhibition of Des1 activity in T98G cell lysates by AL-088 and AL-274. Des1 activity was measured as described above for panel B. Data are expressed as means ± SD (*n* = 3).

Within the sphingolipid biosynthetic pathway (Fig. 6A), the conversion of dhCer to Cer is catalyzed by sphingolipid Δ^4^-desaturase (Des1), which introduces a double bond at C_4_-C_5_ in the sphingosine chain. The next step in sphingolipid synthesis is the conversion of Cer to SM by SM synthases (SMSs). In addition, SMSs can also naturally convert dhCers into dhSMs, these being minor components of cellular SMs (Fig. 6A). Accordingly, we hypothesized that the accumulation of dhSM in cells treated with AL-088 and AL-274 could be due to the blockage of Des1 activity. To test this hypothesis, the effects of both compounds on Des1 activity were evaluated by measuring the conversion of the fluorescent probe *N*-{6-[(7-nitro-2-1,3-benzoxadiazol-4-yl)amino]hexanoyl}-d-*erythro*-dihydrosphingosine (dhCerC6NBD) to CerC6NBD, which was monitored by high-performance liquid chromatography (HPLC) coupled with fluorescence detection (40). Treatment of T98G cell cultures with AL-088 or AL-274 resulted in a reduction in dhCerC6NBD conversion, confirming that these compounds inhibited Des1 (Fig. 6B). Our compounds also inhibited Des1 in cell lysates in a dose-dependent manner, with half-maximal inhibitory concentration (IC_50_) values of 1.0 and 7.8 μM for AL-088 and AL-274, respectively (Fig. 6C). Overall, these results demonstrate that the synthetic polyphenols AL-088 and AL-274 inhibit Des1 activity.

### Dihydrosphingomyelin inhibits WNV infection.

**FIG 7:**
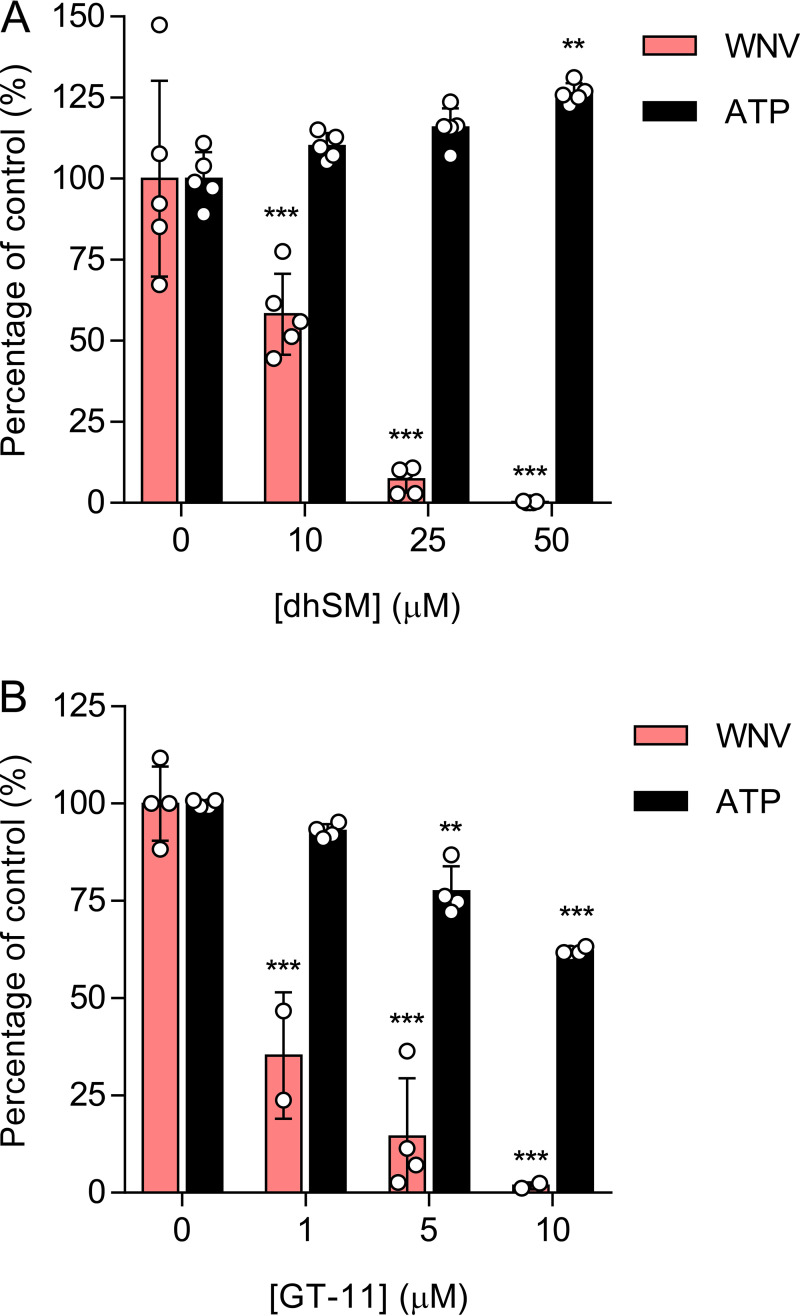
Dihydrosphingomyelin inhibits WNV infection. (A) Antiviral activity of dhSM against WNV. Vero cells were loaded with dhSM for 24 h and infected with WNV (MOI of 1 PFU/cell), and the virus yield in the supernatant was determined at 24 h p.i. The cytotoxicity of the compounds was measured by the determination of the cellular ATP concentration in uninfected samples. Data are expressed as means ± SD (*n* = 5). Each dot denotes a single biological replicate. **, *P < *0.01; ***, *P < *0.001 (by ANOVA and Student’s *t* test using Bonferroni’s correction). (B) Antiviral activity of the Des1 inhibitor GT-11 against WNV. Vero cells were treated with GT-11 and infected as described above for panel A. Data are expressed as means ± SD (*n* = 4 to 5). Each dot denotes a single biological replicate. **, *P < *0.01; ***, *P < *0.001 (by ANOVA and Student’s *t* test using Bonferroni’s correction).

The analysis of the lipidomic data pointed to the increase in dhSM as the putative mechanism behind the antiviral activity of AL-088 and AL-274. Thus, the direct effect of this lipid on WNV infection was evaluated (Fig. 7A and Table S1). The addition of exogenous dhSM significantly reduced WNV infection in a dose-dependent manner, without reducing cell viability estimated by ATP measurements. This result suggests that the antiviral effects of AL-088 and AL-274 are related to Des1 inhibition leading to the accumulation of dhSM. To further explore the potential of Des1 as a novel druggable target against WNV, the effect of the structurally unrelated Des1 inhibitor GT-11 {*N*-[(1*R*,2*S*)-2-hydroxy-1-hydroxymethyl-2-(2-tridecyl-1-cyclopropenyl)ethyl]octanamide} (41) was studied (Fig. 7B and Table S1). Interestingly, GT-11 inhibited WNV infection in a dose-dependent manner, confirming that the pharmacological inhibition of Des1 reduced WNV infection. Overall, these results demonstrate that an increase in the dhSM level, either by adding it exogenously or by inhibiting Des1, hampers WNV replication. Moreover, these results support that Des1 is a suitable druggable target for antiviral intervention against WNV.

## DISCUSSION

It is well established that the amphipathic character of polyphenols, with hydrophobic aromatic rings and hydrophilic hydroxyl groups, plays a major role in their biological activities (42, 43). The incorporation of polymethylene chains may further affect this amphipathic character, which can additionally be modified by the functionalization of the distal part of the spacer with a second phenolic unit (18). Indeed, this approach has led in our hands to compounds with significant antiviral activity against HCV, probably affecting lipid metabolism (18). This has now been studied in detail using WNV as a clinically relevant example of a flavivirus. Based on the structure-activity relationship data included in Results, compounds AL-088 and AL-274 afforded the best selectivity indices (>45 and >416, respectively), with EC_50_ values in the low- or submicromolar range against WNV (EC_50_s of 2.2 and 0.24 μM, respectively, in Vero cells; EC_50_s of 2.2 and 1.9 μM, respectively, in SH-SY5Y cells). Moreover, both compounds also effectively inhibited the multiplication of other flaviviruses, namely, USUV, ZIKV, and DENV-2, in all cases with EC_50_ values of <10 μM. Interestingly, these compounds showed a high degree of specificity for flaviviruses compared to other RNA viruses, exhibiting lower antiviral activity against the rhabdovirus VSV, while no antiviral activity was observed against the picornavirus CVB5.

The analysis of the lipidome of Vero cells treated with AL-088 and AL-274 revealed significant changes compared to untreated cells. Particularly, a significant increase in the level of dhSMs (d18:0/18:0, d18:0/22:0, d18:0/24:0, and d18:0/24:1) was detected, which led us to hypothesize that the accumulation of dhSM could account for the antiviral effect observed with both compounds. Supporting our hypothesis, the addition of external dhSM also reduced WNV infection. Moreover, when both compounds were tested as Des1 inhibitors in cells or cell lysates, dose-dependent inhibition was detected. Indeed, similar antiviral effects were observed for treatment with the validated Des1 inhibitor GT-11. Dihydrosphingolipids, which were largely ignored since no direct biological activities could be ascribed to them and whose tissue concentrations are significantly lower than those of sphingolipids, are receiving increased consideration due to their important roles in metabolic pathways and cell signaling networks (44). Their physiological roles involve, among others, responses to cellular stress and autophagy, prodeath and prosurvival pathways, hypoxia, or immune responses (45). However, to our knowledge, the role of dhSM in viral infections was described only for retroviruses some years ago (46–48). Key enzymes in dhSM metabolism are dhCer desaturases, with two isoforms: Des1, which is ubiquitously distributed, and Des2, which is expressed in the intestine, skin, and kidney (49). A number of compounds have been described as Des1 inhibitors, which include sphingolipid analogues such as GT-11 but also quite a variety of chemical structures such as fenretinide {also designated 4-HPR [*N*-(4-hydroxyphenyl)retinamide]}, Δ^9^-tetrahydrocannabinol (THC), the sphingosine kinase 1/2 inhibitor (SKI-II), or resveratrol (49). Even more, unraveling the mechanism of action of certain anticancer drugs such as ABTL0812 has shown that, at least in part, their mechanism of action to induce autophagy is related to their capacity to inhibit Des1, leading to the accumulation of dhCer (50). While for GT-11, the mechanism of action involves competition with the natural substrate (41), for many other compounds, the exact mechanism of action against Des1 has not been unequivocally elucidated. As an example, fenretinide and SKI-II, which contain a 4-aminophenol in their structure, have been proposed to generate reactive iminoquinones that might irreversibly react with nucleophilic residues of the protein (51). Moreover, the Fe_2_O_3_ in the active site of Des1 may contribute to the oxidation of the inhibitors (51). Thus, the ability of the galloyl derivatives AL-088 and AL-274 to inhibit Des1 activity, in both intact cells and cell lysates, suggests that inhibition can occur at the protein level and/or by interaction with the Des1-associated electron transport chain, as reported previously for other phenolic compounds such as fenretinide, resveratrol, or Δ^9^-tetrahydrocannabinol (49). Although AL-088 and AL-274 exhibited low-cytotoxicity profiles in Vero cells, these compounds were more cytotoxic in SH-SY5Y neuroblastoma cultures, which is consistent with previous reports on growth arrest and cytotoxicity upon Des1 inhibition in cancer cells (50, 52).

The data presented here show that treatment with AL-088 or AL-274 leads to the accumulation of dhSM in the host cell, which reduces infection by WNV. Therefore, the accumulation of dhSM in cells treated with AL-088 and AL-274 may lead to changes in the membrane properties that result in the impairment of viral infection, including the inhibition of viral entry, as described previously for human immunodeficiency virus type 1 (48), or the assembly of replication complexes and particle biogenesis. The accumulation of dhSMs has been proposed to involve the rigidification of membranes by effectively forming ordered domains through their high potential to induce intermolecular hydrogen bonds (53, 54). This mechanism of action would be consistent with the results of previous studies indicating that flaviviruses are highly dependent on sphingolipid metabolism and that perturbation of these pathways affects infection by targeting replication and morphogenesis (32–34, 55). In fact, the replication and biogenesis of flaviviruses take place coupled with highly remodeled cytoplasmic membranous structures derived from the endoplasmic reticulum (ER) that have a specific lipid composition (34). As these polyphenols also exerted antiviral activity against an HCV replicon system, these would further support their ability to interfere with the replication stages of *Flaviviridae* (18). Thus, it is reasonable to propose that the accumulation of dhSM may result in the stiffening of the cellular membranes and that this would severely affect multiple steps of WNV infection. The antiviral activity of AL-088 and AL-274 exerted against VSV is also consistent with previous reports indicating that sphingolipids may account for multiple roles during VSV infection (56–58).

Our work illustrates the importance of Des1 inhibitors as host-directed antiviral agents, reveals the crucial role of dhSMs in flavivirus infection, and contributes to expanding the portfolio of the roles and functions of dihydrosphingolipids beyond their implication in cancer and metabolic diseases. The ability of these polyphenols to inhibit infection by medically relevant flaviviruses makes them promising leads for the future development of antiviral therapies.

## MATERIALS AND METHODS

### Chemical compounds.

The synthesis of the polyphenols used in this study has been described previously by us (18). *N*-[(1*R*,2*S*)-2-Hydroxy-1-hydroxymethyl-2-(2-tridecyl-1-cyclopropenyl)ethyl]octanamide (GT-11) was synthesized as described previously (41). Stock solutions of polyphenols and GT-11 were prepared at 10 mM in dimethyl sulfoxide (DMSO). *N*-Lauroyl-d-*erythro*-sphinganylphosphorylcholine, a synthetic dhSM, was purchased from Avanti Polar Lipids Inc. (Birmingham, AL) and dissolved in ethanol to create a 50 mM stock solution. *N*-{6-[(7-Nitro-2-1,3-benzoxadiazol-4-yl)amino]hexanoyl}-d-*erythro*-dihydrosphingosine (dhCerC6NBD) was synthesized as described previously (59). The same concentration of the drug solvent (DMSO in the case of polyphenols and GT-11 or ethanol in the case of dhSM) was used as the drug vehicle.

### Viruses and infections.

Virus infections were performed on Vero ATCC CCL-81 or SH-SY5Y ATCC CRL-2266 cells (ATCC, Manassas, VA). Vero cells were cultured in minimal essential medium (MEM) (Corning, Manassas, VA) supplemented with 10% fetal bovine serum (Gibco, Life Technologies, Paisley, UK), and SH-SY5Y cells were grown in Dulbecco’s modified Eagle’s medium (DMEM)–F-12 medium (Biowest, Nuaillé, France) supplemented with 10% fetal bovine serum. A penicillin-streptomycin mixture (Corning) and l-glutamine (Corning) were also added to cell cultures. WNV New York 99 (60), USUV SAAR 1776 (60), American ZIKV PA259459 (61), a cell-passaged derivative of DENV-2 16681 (16), VSV Indiana (61), and CVB5 Faulkner (62) were used. Procedures for infections in liquid medium and virus titrations in Vero cells in semisolid agar medium were previously described (62, 63). WNV, USUV, ZIKV, VSV, and CVB5 titers were determined at 24 h postinfection (p.i.). DENV-2 titers were determined at 48 h p.i. A multiplicity of infection (MOI) of 1 PFU/cell was used for all experiments unless otherwise indicated.

### Cellular toxicity.

Cell viability was measured in uninfected cells by ATP quantification using the CellTiter Glo luminescent cell viability assay (Promega, Madison, WI). Proliferation was measured after 24 h of treatment of uninfected cells plated at a low density (<50% confluence) by the determination of the cell number by the use of a TC20 automated cell counter (Bio-Rad, Hercules, CA) and trypan blue dye (Bio-Rad).

### Reporter virus particles.

WNV single-round reporter virus particles (RVPs) (39) were produced by the complementation in *trans* of a subgenomic reporter replicon, including green fluorescent protein (GFP) (kindly provided by T. C. Pierson), with WNV structural proteins using a pcDNA 3.1(+) expression vector that encoded the prM and E proteins of WNV Novi Sad/12 (GenBank accession number KC407673.1). Plasmids were transfected into human embryonic kidney HEK 293T cells (ATCC CRL-11268) with DharmaFECT kb DNA transfection reagent (Dharmacon, Lafayette, CO). The RVPs in the supernatant were harvested at 48 h posttransfection and used to infect Vero cells. The number of Vero cells expressing GFP was determined by flow cytometry using a FACSCanto II instrument (BD Biosciences, Erembodegem, Belgium) at 48 h p.i.

### Specific infectivity.

Specific infectivity was calculated as the ratio of PFU determined by virus titration to PFU equivalents determined by quantitative reverse transcription-PCR (RT-PCR) by comparison with previously titrated standards. RNA extraction and one-step reverse transcription coupled to quantitative PCR were performed as described previously (30).

### Immunofluorescence and confocal microscopy.

Mouse monoclonal antibody J2 against double-stranded RNA (dsRNA) was obtained from Scicons (Budapest, Hungary). Mouse monoclonal antibody MAB8150 against the WNV E glycoprotein was obtained from EMD Millipore (Billerica, MA). Alexa Fluor 488-labeled goat anti-mouse IgG(H+L) and TO-PRO-3 were obtained from Invitrogen. Immunofluorescence and confocal microscopy were performed as described previously (62).

### Lipidomics.

Lipid extraction, identification, and quantification by mass spectrometry of Vero cells (10^6^ cells/determination) treated with the inhibitors (10 μM for 24 h) or the drug vehicle were performed as described previously (32, 64). The fold change in lipid levels between control and treated cells was calculated as log_2_(treated/control). A principal-component analysis (PCA) was performed using Metaboanalyst 5.0 (65).

### Dihydroceramide desaturase activity.

Desaturase activity was measured in intact T98 cells or lysates after 4 h of incubation with the polyphenols by quantification of the conversion of the dhCerC6NBD probe to the CerC6NBD substrate by HPLC coupled with fluorescence detection (40).

### Data analysis.

Data are presented as means ± standard deviations (SD). The numbers of independent biological replicates analyzed are indicated in the figure legends. Prism 7 for Windows (GraphPad Software, San Diego, CA) was used for statistical analyses. Dose-response curves were calculated by adjusting the sigmoidal log (inhibitor)-versus-normalized response (variable slope) or linear regression in the case of CVB5. Means were compared using one-way analysis of variance (ANOVA) corrected for multiple comparisons with Bonferroni’s correction for pairwise comparisons of multiple treatment groups with a single control group. In the case of lipidomic analyses, comparisons were performed using multiple Student *t* tests with Sidak-Bonferroni correction for lipid subclasses and the false discovery rate (FDR) for lipid species. Significantly altered lipids were considered when the *P* value was <0.05 and the log_2_ fold change over the vehicle was >1.5. Statistically significant differences are denoted in the figures by asterisks (*, *P < *0.05; **, *P < *0.01; ***, *P < *0.001).
