# Supplementary material for: Pharmacological Elevation of Cellular Dihydrosphingomyelin Provides a Novel Antiviral Strategy against West Nile Virus Infection
Source: Antimicrob Agents Chemother. 2023 Mar 15;67(4):e01687-22. doi: 10.1128/aac.01687-22 (PMC10112131; doi:10.1128/aac.01687-22)
Supplement: Supplemental file 1 — Supplemental material. Download aac.01687-22-s0001.pdf, PDF file, 0.5 MB [file aac.01687-22-s0001.pdf]

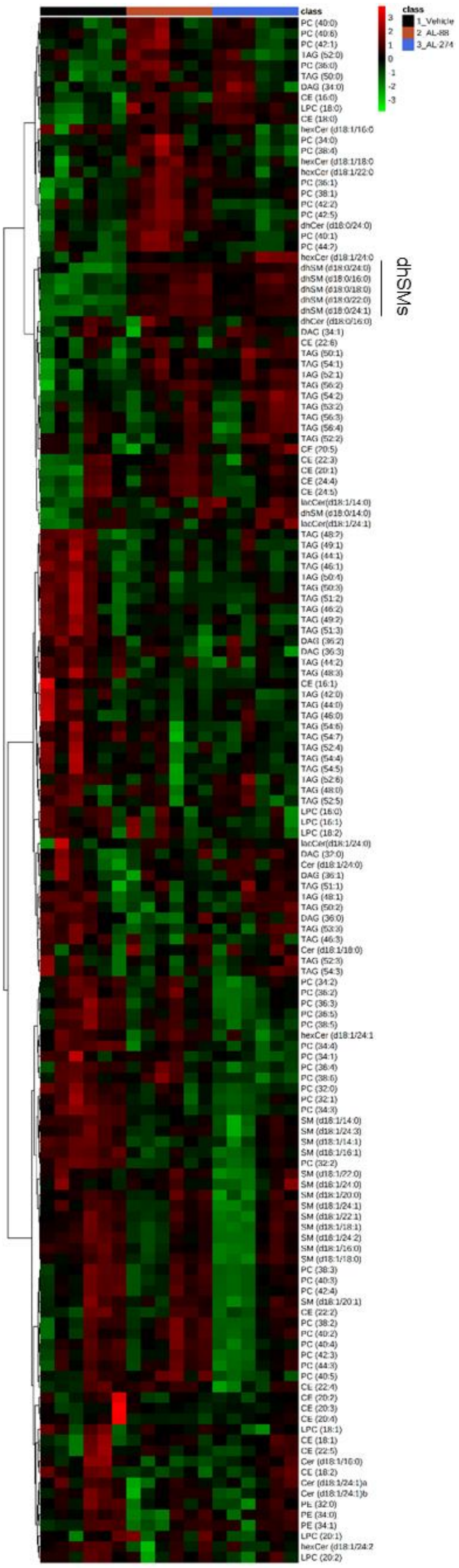

FIG S1 Changes in the lipidome of cells treated with AL-088 and AL-274. Vero cells were treated with 10 µM AL-088, 10 µM AL-274 or vehicle (DMSO) for 24 h. Lipids were identified, and quantified by mass spectrometry as described in Materials and Methods. The heatmap was created using Metaboanalyst 5.0. Data from the 6 biological replicates for each condition are displayed (n = 6).

TABLE S1 Mean virus yield obtained in the experiments displayed in the figures expressed as PFU/mL.

Fig1B	[comp.](μM)	Gallic acid	AL-071	AL-072	AL-085	AL-088	AL-274				
	0.001	5.72E+08	1.12E+08	1.31E+08	1.40E+08	2.70E+08	2.43E+08				
	0.01	5.07E+08	1.02E+08	1.32E+08	1.02E+08	2.75E+08	1.85E+08				
	0.1	6.10E+08	1.10E+08	1.00E+08	9.07E+07	3.14E+08	1.33E+08				
	1	5.47E+08	8.17E+07	1.29E+08	4.33E+07	1.79E+08	1.16E+08				
	10	5.73E+08	5.97E+03	7.26E+06	<50	5.35E+07	2.35E+07				
	25	5.48E+08	<50	3.02E+02	<50	1.14E+07	1.42E+07				
	50	4.62E+08	<50	<50	<50	2.01E+06	9.10E+05				
	100	2.93E+08	<50	<50	<50	1.30E+05	1.25E+05				
**Fig1D**	**[comp.](μM)**	**AL-088 MOI 0.1**	**AL-088 MOI 1**	**AL-088 MOI 10**	**AL-274 MOI 0.1**	**AL-274 MOI 1**	**AL-274 MOI 10**				
	0	1.08E+08	6.92E+08	1.75E+09	1.35E+08	5.17E+08	1.22E+09				
	10	1.73E+07	8.04E+07	2.14E+08	8.45E+06	7.88E+07	2.47E+08				
	50	4.70E+05	2.33E+06	1.16E+07	5.63E+05	3.20E+06	1.71E+07				
**Fig2**	**[comp.](μM)**	**AL-088**	**AL-274**								
	0.001	1.10E+06	1.57E+06								
	0.01	1.00E+06	1.57E+06								
	0.1	6.83E+05	1.39E+06								
	1	1.24E+06	1.26E+06								
	10	1.25E+03	3.58E+03								
	25	2.57E+01	<50								
	50	<50	<50								
	100	<50	<50								
**Fig3**	**[comp.](μM)**	**AL-088 USUV**	**AL-088 ZIKV**	**AL-088 DENV-2**	**AL-088 VSV**	**AL-088 CVB5**	**AL-274 USUV**	**AL-274 ZIKV**	**AL-274 DENV-2**	**AL-274 VSV**	**AL-274 CVB5**
	0.001	6.25E+06	4.01E+06	1.17E+07	9.97E+08	8.58E+07	1.28E+06	2.96E+06	7.57E+06	8.28E+08	9.25E+07
	0.01		4.56E+06	1.14E+07	1.21E+09	7.12E+07	1.19E+06	2.87E+06	7.89E+06	8.40E+08	7.64E+07
	0.1	4.33E+06	3.05E+06	8.35E+06	1.03E+09	6.85E+07	1.26E+06	3.19E+06	6.88E+06	9.76E+08	8.20E+07
	1	3.64E+06	4.18E+06	9.61E+06	9.59E+08	8.01E+07	7.44E+05	2.25E+06	7.68E+06	9.10E+08	8.13E+07
	10	7.38E+05	3.46E+05	6.34E+06	8.72E+08	6.45E+07	1.11E+05	2.62E+05	3.29E+06	1.27E+09	8.84E+07
	25		1.44E+04	2.12E+06	4.07E+08	7.26E+07		1.41E+04	2.88E+05	7.33E+08	6.70E+07
	50		1.13E+03	4.05E+05	2.22E+08	7.43E+07		2.81E+04	4.46E+03	2.70E+08	7.30E+07
	100	<50	<50	2.24E+04	1.66E+08	8.33E+07	<50	<50	1.85E+02	1.06E+08	7.92E+07
**Fig4C**	**[comp.](μM)**	**AL-088 1 hpi**	**AL-088 3h pi**								
	0	1.02E+08	1.07E+08								
	10	4.79E+07	4.06E+07								
	50	9.95E+05	1.05E+06								
**Fig4D**	**[comp.] μM)**	**AL-274 1 hpi**	**AL-274 3h pi**								
	0	6.21E+07	7.33E+07								
	10	1.68E+07	1.13E+07								
	50	1.36E+06	1.39E+06								
**Fig7A**	**[comp.](μM)**	**dhSM**									
	0	1.97E+08									
	10	1.17E+08									
	25	2.01E+07									
	50	1.72E+05									
**Fig7B**	**[comp.](μM)**	**GT-11**									
	0	2.53E+08									
	1	8.15E+07									
	5	2.14E+07									
	10	4.19E+06									
